# Understanding Anti-Obesity Potential of Four Porphyrin Compounds by Investigating Pancreatic Lipase Inhibition

**DOI:** 10.3390/molecules30132701

**Published:** 2025-06-23

**Authors:** Jie Zhou, Xinrui Wang, Yangyuxin Liu, Xiaochen Li, Mingze Huang, Helong Bai, Jingang Mo

**Affiliations:** College of Chemistry, Changchun Normal University, Changchun 130032, China; zhoujie001119@163.com (J.Z.); xrznba666@163.com (X.W.); lyyx24@outlook.com (Y.L.); 17538647968@163.com (X.L.); asdcfb@126.com (M.H.)

**Keywords:** porphyrin compound, pancreatic lipase, inhibition

## Abstract

Obesity is a chronic epidemic caused by abnormal fat metabolism. As a key digestive enzyme, pancreatic lipase (PL) is an important target for regulating fat metabolism. The inhibitory potential of 5,10,15,20-Tetrakis (4-aminophenyl) porphyrin (TAPP), 5,10,15,20-Tetrakis (4-hydroxyphenyl) porphyrin (THPP), meso-Tetra (4-carboxyphenyl) porphine (TCPP), Cu (II) meso-Tetra (4-carboxyphenyl) porphine (Cu-TCPP) on PL was studied by enzymatic kinetics, multi-spectral, and molecular simulation technology. THPP, TCPP, TAPP, and Cu-TCPP all had good PL inhibitory activity (IC_50_ range: 97.49–248.70 μM) and were uncompetitive inhibitors. The order of inhibitory ability was: THPP > TCPP > TAPP > Cu-TCPP. The fluorescence quenching mechanism of THPP to PL was a mixed quenching dominated by static quenching, while TCPP, TAPP, and Cu-TCPP were static quenching. The binding of THPP, TCPP and TAPP to PL was mainly driven by hydrogen bonds and van der Waals forces, while Cu-TCPP was mainly driven by a hydrophobic interaction. Four porphyrin compounds changed the conformation of PL, affected the microenvironment of Tyr and Trp residues, and induced changes in the secondary structure of PL, thereby reducing the stability and catalytic activity of PL. Hydrogen bonds played an important role in the binding stability of THPP, TCPP, TAPP, and PL.

## 1. Introduction

**Figure 1:**
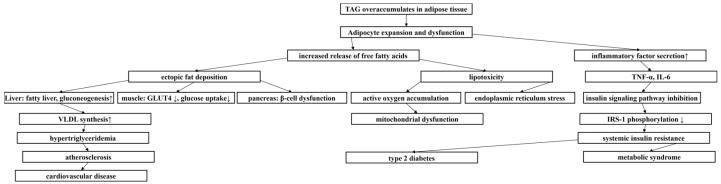
Metabolic cascade triggered by TAG accumulation. ↑ represented increase, ↓ represented decrease.

Obesity, as a nutritional metabolic disorder [1], has been listed as a global health crisis by the WHO [2], with its escalating prevalence highlighting the urgency for the prevention and treatment of obesity. The pathological features of obesity are characterized by abnormally elevated levels of triacylglycerol (TAG) in plasma (Figure 1), resulting in excessive accumulation and abnormal distribution of adipose tissue [3], and then overweight. Body mass index (BMI) is the most commonly used powerful indicator to evaluate the degree of overall obesity. A BMI of 24.0–27.9 kg/m^2^ is defined as overweight, and BMI of 28.0 kg/m^2^ or higher is defined as obesity. Waist circumference (WC) is a simple index to determine abdominal visceral obesity. The defined parameters of Chinese adults are male waist circumference of 90 cm and female waist circumference of 85 cm [4]. Visceral adiposity index (VAI) is an obesity index that can reflect visceral fat function. The formation and development of obesity involves the combined effects of multiple factors such as heredity, social environment, and lifestyle. In particular, the lifestyle of sedentary and excessive energy-intensive food intake makes food-borne obesity extremely prominent [5], which promotes the rapid growth of the incidence of obesity, a chronic metabolic disease. Obesity is not only a major risk factor for stroke, cardiovascular disease, and a variety of malignant tumors (such as esophageal adenocarcinoma and colorectal cancer), but it is also closely related to metabolic syndrome [6]. The high prevalence of obesity not only poses a serious challenge to global healthcare systems, but it has also become an urgent economic and social problem to be solved.

The identification and characterization of several gastrointestinal enzymes involved in nutrient digestion provide potential targets for the treatment of obesity [7]. Approximately 90–95% of dietary fat exists in the form of TAG [8]. The digestion process begins with initial hydrolysis by tongue lipase in the oral cavity and then is hydrolyzed by gastric lipase in the stomach and then is finally hydrolyzed into monoglycerides and free fatty acids [9] under the synergistic effect of pancreatic lipase (PL) and coenzyme in the duodenum. After the digestion product is absorbed by intestinal epithelial cells, and TAG is re-synthesized, chylomicrons are formed and transported to various tissues of the body for tissue utilization or storage [10]. PL (triacylglycerol lipase) is an important energy metabolism enzyme [11] for the digestion and absorption of dietary lipids in the small intestine. It is responsible for hydrolyzing 50–70% [12] of dietary TAG.

When the body reaches a negative energy balance, that is, energy absorption is less than energy expenditure [13], it will lead to weight loss. However, it is often difficult to achieve the desired results by relying solely on the combination of diet control and exercise. Therefore, other forms of obesity treatment strategies, especially drug therapy [14], have attracted increasing attention. At present, anti-obesity drugs are mainly divided into two major categories: one acts on the central nervous system [15] to suppress appetite or promote hormone secretion to increase energy consumption to achieve the purpose of treatment; the other is PL inhibitors. The inhibition of PL has become the most widely studied mode of action in the field of anti-obesity drug development [16]. Inhibition of PL can retard the digestion and absorption of nutrients, thereby inhibiting the absorption of dietary lipids and achieving the purpose of controlling and treating obesity. Common natural active compounds as PL inhibitors mainly include polyphenols, flavonoids, saponins, terpenoids, alkaloids, etc. [17]. Orlistat is the only lipase inhibitor [18] approved by FDA for obesity treatment, and it is one of the best-selling anti-obesity drugs on the market [19]. Orlistat is a semi-synthetic hydrogenated derivative of lipostatin [20]. As a long-acting and efficient PL inhibitor, it irreversibly inhibits the enzyme’s catalytic activity by forming a covalent bond [21] with the serine residue [22] of the active center of PL. This inhibitory effect blocks the hydrolysis of dietary TAG, so that they cannot be converted into absorbable monoacylglycerols and free fatty acids. Because the intestinal tract cannot absorb undigested TAG, the effect of weight loss is achieved. Clinical studies demonstrate that long-term use of orlistat can suppress appetite, reduce weight and reduce intra-abdominal fat accumulation, but its side effects are unavoidable. Common adverse reactions include gastrointestinal symptoms [23] such as steatorrhea, oily stools, and fecal spots, and liver symptoms [24] such as cholelithiasis, biliary hepatitis, and subacute liver failure. Therefore, it is of great significance to develop efficient, safe, and widely available PL inhibitors.

**Figure 2:**
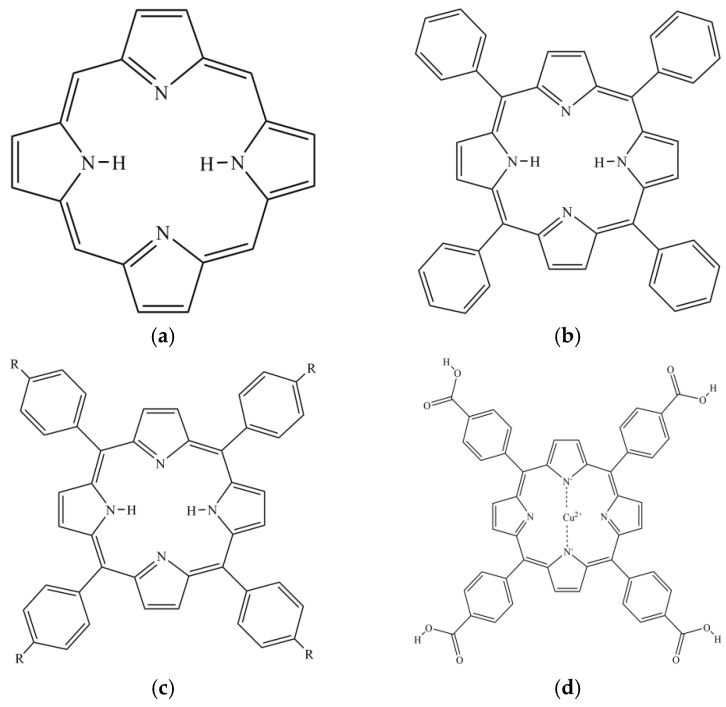
Structural formula of (**a**) Porphyrin; (**b**) TPP; (**c**) TAPP: R = NH_2_, THPP: R = OH, TCPP: R = COOH; (**d**) Cu-TCPP.

Porphyrin is a highly conjugated aromatic macrocyclic compound [25] formed by four pyrrole rings bridged by methylene (=CH-) (Figure 2). Porphyrin compounds are widely found in nature and organisms [26]. Heme in animals is an iron porphyrin complex containing coordination oxygen [27], which bears the physiological function of catalyzing and transporting oxygen carrier. Hemocyanin (copper porphyrin) performs the physiological function of oxygen carrier. Chlorophyll (magnesium porphyrin) and vitamin B_12_ (cobalt porphyrin) in plants are involved in important life processes. Porphyrins play vital roles in numerous biological processes and assume diverse physiological functions [28,29], so they are often referred to as ‘pigments of life’. With unique structure and chemical properties, porphyrin compounds have attracted much attention in the field of medicine, especially as a new class of anticancer drugs. Porphyrin compounds are also expected to have other biological activities, such as the inhibition of pancreatic lipase activity. Tetraphenylporphyrin (TPP) is an aromatic macrocyclic compound formed by porphyrin as the parent and benzene ring replacing four meso-hydrogen atoms (Figure 2). The porphyrin ring and benzene ring of TPP together provide stronger hydrophobicity and a larger conjugated system, which makes it have good biocompatibility and remarkable structural stability.

The amino, hydroxyl, and carboxyl groups of functional groups were modified to the benzene ring of TPP to obtain better derivatives 5,10,15,20-Tetrakis (4-aminophenyl) porphyrin (TAPP), 5,10,15,20-Tetrakis (4-hydroxyphenyl) porphyrin (THPP), meso-Tetra (4-carboxyphenyl) porphine (TCPP) (Figure 2). The amino (-NH_2_) and hydroxyl (-OH) groups are electron-donating groups, which increase the electron density of the porphyrin macrocycle. This also endows TAPP with high hydrophilicity and reactivity, and endows THPP with good solubility and excellent light capture ability. The carboxyl (-COOH) group is an electron-withdrawing group, which reduces the electron density of the porphyrin macrocycle and endows TCPP with a strong coordination ability and chemical stability. Therefore, based on the excellent coordination ability of TCPP, copper ions were introduced to form Cu (II) meso-Tetra (4-carboxyphenyl) porphine (Cu-TCPP, Figure 2). Copper is one of the transition metals found in the heart, liver, lung, and gallbladder [30]. It plays an important role in electron transfer, iron metabolism, immune regulation, and glucose metabolism. Numerous studies have demonstrated the different pharmacological effects of copper complexes, including anti-diabetes, anti-oxidation, anticancer and anti-ulcer activities. Peng et al. [31] found that Hsp-Cu (II) showed stronger inhibitory ability on glycoside hydrolase than hesperetin (Hsp). The IC_50_ value of Hsp-Cu (II) inhibiting α-amylase was 60.30 ± 0.90 μM, which was lower than that of Hsp (115.60 ± 1.10 μM). The IC_50_ value of inhibiting α-glucosidase was 1.25 ± 0.03 μM, which was lower than that of Hsp (55.20 ± 0.10 μM).

## 2. Results

### 2.1. Pancreatic Lipase Inhibition by Four Porphyrin Compounds

**Figure 3:**
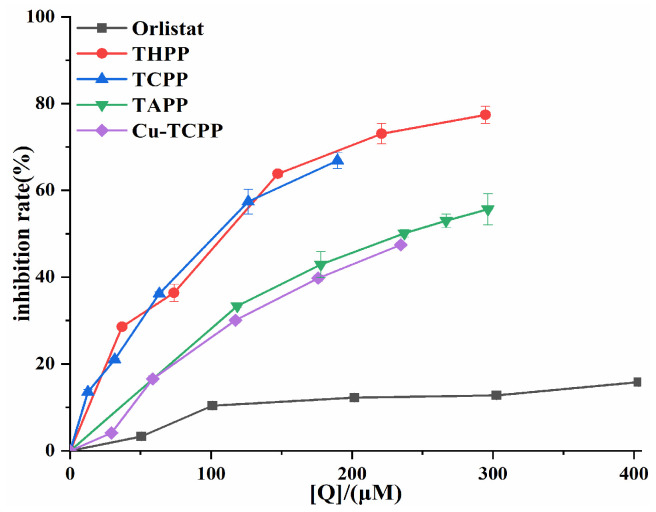
PL inhibition by four porphyrin compounds and orlistat.

An in vitro PL activity assay system was established to investigate the inhibitory ability of four porphyrin compounds on PL. THPP, TCPP, TAPP, and Cu-TCPP all had good inhibitory ability of PL activity (Figure 3). The inhibition rates of four porphyrin compounds on PL increased with the increase in concentration in the experimental concentration range. The magnitude of PL inhibition by four porphyrin compounds and positive control orlistat at the same concentration was THPP (IC_50_ = 97.49 μM) > TCPP (IC_50_ = 100.10 μM) > TAPP (IC_50_ = 236.40 μM) > Cu-TCPP (IC_50_ = 248.70 μM) > Orlistat (IC_50_ = 6.25 mM).

### 2.2. Mode of Inhibition of Pancreatic Lipase by Four Porphyrin Compounds

**Figure 4:**
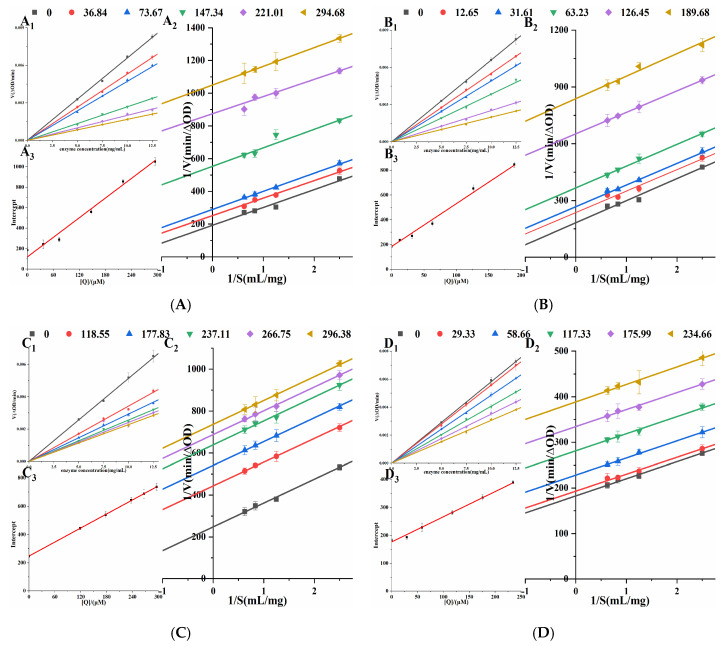
Mode of inhibition studies on PL. (**A**) THPP (**B**) TCPP (**C**) TAPP (**D**) Cu-TCPP (**A_1_**–**D_1_**) respective Reversibility effect. (**A_2_**–**D_2_**) respective Lineweaver-Burk plot. (**A_3_**–**D_3_**) respective Intercept quadratic plot.

Orlistat irreversibly inhibits the enzyme’s catalytic activity by forming a covalent bond [21] with SER152 of the active center of PL. In Figure 4, under different concentrations of four porphyrin compounds, all the straight lines passed through the origin, and the slope of the straight line decreased with the increase in the concentrations. Therefore, the inhibitions of THPP, TCPP, TAPP, and Cu-TCPP on PL activity were reversible. Lineweaver-Burk double reciprocal plots were constructed with 1/*V* to 1/*S* (Figure 4). The straight lines of four porphyrin compounds were parallel to each other, indicating that they were typical uncompetitive PL inhibitors. For uncompetitive inhibition (UC), the kinetic equation is as in Formula (1). Table 1 showed that with the increase in the concentrations of four porphyrin compounds, *K_m_* gradually decreased and *V_max_* gradually decreased. Therefore, the inhibitory effects of THPP, TCPP, TAPP, and Cu-TCPP on PL were UC. Plotting the concentrations against the intercepts of the straight lines showed a good linear fit, indicating that four porphyrin compounds had only one or a single inhibition site on PL. *K_i_* was calculated by Formula (2). The smaller *K_i_* is, the stronger the binding ability of the inhibitor to the enzyme, and the stronger the inhibition ability of the enzyme. The inhibition constants *K_i_* of four porphyrin compounds on PL were THPP, TCPP, TAPP, and Cu-TCPP in descending order, which was the same as the order of IC_50_ value, proving that THPP had the strongest inhibitory effect on PL, followed by TCPP, TAPP, and Cu-TCPP.

The UC formula is as follows:

(1) $$\frac{1}{v}=\frac{K_{m}}{V_{max}}\frac{1}{\left[S\right]}+\frac{1}{V_{max}}\left(1+\frac{\left[I\right]}{K_{i}}\right),$$

(2) $$Y-\mathrm{int}ercept=\frac{1}{V_{max}}+\frac{\left[I\right]}{V_{max}K_{i}},$$

In the formula, v, *V_max_*, *K_m_*, and *K_i_* represent reaction rate, maximum reaction rate, Michaelis constant, and free enzyme inhibition constant, respectively. [S] denotes the substrate concentration. [I] denotes the inhibitor concentration.

### 2.3. Analysis of in Vitro Stability

**Figure 5:**
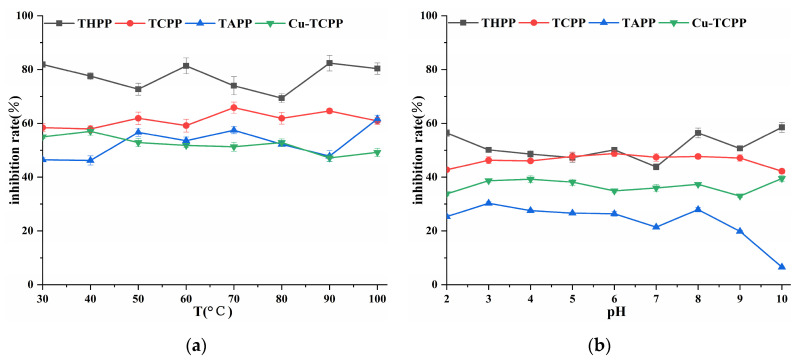
(**a**) Temperature stability; (**b**) Acid-base stability.

Four porphyrin compounds showed no significant change in the inhibition rate of PL in the temperature range of 30~100 °C (Figure 5), which indicated that they had good thermal stability in a short period of time. THPP, TCPP, and Cu-TCPP had a wide range of pH tolerance, and the inhibitory activity changed little between pH 2.0~10.0 (Figure 5). The inhibitory activity of TAPP changed little between pH 2.0~9.0. Four porphyrin compounds had higher inhibitory activity under weak alkaline conditions than under acidic conditions. The inhibitory activity of temperature treatment was higher than that of acid-base treatment at the same concentration.

### 2.4. Fluorescent Quenching and Binding Interactions of Pancreatic Lipase with Four Porphyrin Compounds

**Figure 6:**
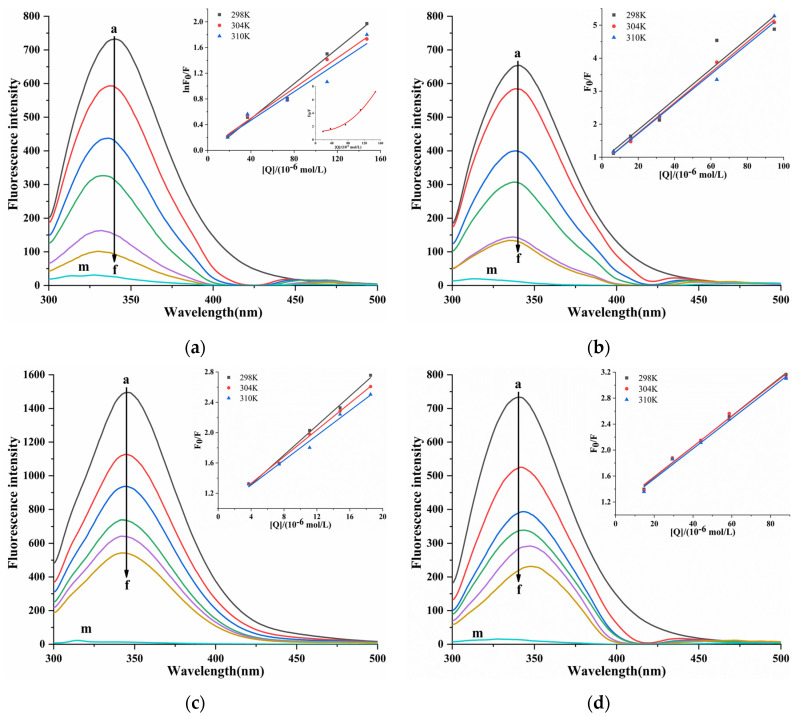
Fluorescent emission spectra and Stern-Volmer plots for fluorescent quenching of PL by (**a**) THPP (**b**) TCPP (**c**) TAPP (**d**) Cu-TCPP. (**a**) c(THPP) = 0, 18.42, 36.84, 73.67, 110.51, 147.34 μM; (**b**) c(TCPP) = 0, 6.32, 15.81, 31.61, 63.23, 94.84 μM; (**c**) c(TAPP) = 0, 3.70, 7.41, 11.11, 14.82, 18.52 μM; (**d**) c(Cu-TCPP) = 0, 14.67, 29.33, 44.00, 58.66, 88.00 μM; for Curves a→f, respectively. Curve m showed the emission spectra of THPP, TCPP, TAPP, Cu-TCPP only.

The fluorescence spectra of the interaction between four porphyrin compounds and PL (Figure 6). With the addition of four porphyrin compounds, the fluorescence intensity of PL gradually decreased, indicating that four porphyrin compounds quenched the endogenous fluorescence of PL. With the increase in the concentration of THPP, TCPP, and TAPP, the emission wavelength of PL was blue-shifted, and THPP caused the largest blue-shift, followed by TCPP and TAPP, indicating that THPP, TCPP, and TAPP reduced the polarity of the amino acid residue microenvironment of PL and increased the hydrophobicity, which was usually related to protein folding. With the increase in the concentration of Cu-TCPP, the emission wavelength of PL was red-shifted, indicating that the interaction between Cu-TCPP and PL changed the amino acid residue microenvironment of PL. The polarity increased and the hydrophobicity decreased, indicating that the protein may undergo structural unfolding [32].

The quenching mechanism between four porphyrin compounds and PL was determined by the Stern–Volmer equation [33]. In the Stern–Volmer curve of PL-THPP, the *F*_0_*/F* relative to [Q] was an upward curve, which was inclined toward the y-axis [34] (Figure 6), indicating that there was a mixed quenching of static quenching and dynamic quenching between THPP and PL. Therefore, the modified Stern–Volmer Formula (4) was applied to calculate *K_SV_*. In Table 2, it was observed that the *K_SV_* value decreased with increasing temperature, and the *K_q_* value was greater than the maximum diffusion collision quenching constant (2.0 × 10^10^ L/mol/s). It showed that the quenching mechanism of THPP on PL was a mixed quenching dominated by static quenching and supplemented by dynamic quenching. The *F*_0_/*F* of TCPP, TAPP, and Cu-TCPP showed a linear relationship with [Q]. The *K_SV_* and *K_q_* calculated by the Stern–Volmer Formula (3) were summarized in Table 2. It was observed that the *K_SV_* value decreased with increasing temperature, and the *K_q_* value was much larger than 2.0 × 10^10^ L/mol/s. The results showed that the quenching of PL by TCPP, TAPP, and Cu-TCPP was due to the static quenching caused by the formation of complex with PL.

Judged by the Stern–Volmer equation, the formula is referred to (3):

(3) $$\frac{F_{0}}{F}=1+K_{SV}[Q]=1+K_{q}\tau_{0}[Q],$$

The modified Stern–Volmer equation [34], formula reference (4):

(4) $$\frac{F_{0}}{F}=e^{K_{SV}[Q]}=e^{K_{q}\tau_{0}[Q]},$$

The double logarithmic equation:

(5) $$\log\frac{F_{0}-F}{F}=\log K_{a}+n\log[Q],$$

In the formula, *F_0_* and *F* are the fluorescence intensities of the enzyme before and after the addition of four porphyrin compounds. *K_SV_* and *K_q_* represent the fluorescence quenching constant and the quenching rate constant, respectively. τ_0_ (10^−8^ s) is the average lifetime of the fluorescent molecule in the absence of the fluorescence quencher. [Q] denotes the concentration of the porphyrin compound. *K_a_* is the binding constant. *n* is the number of binding sites.

**Figure 7:**
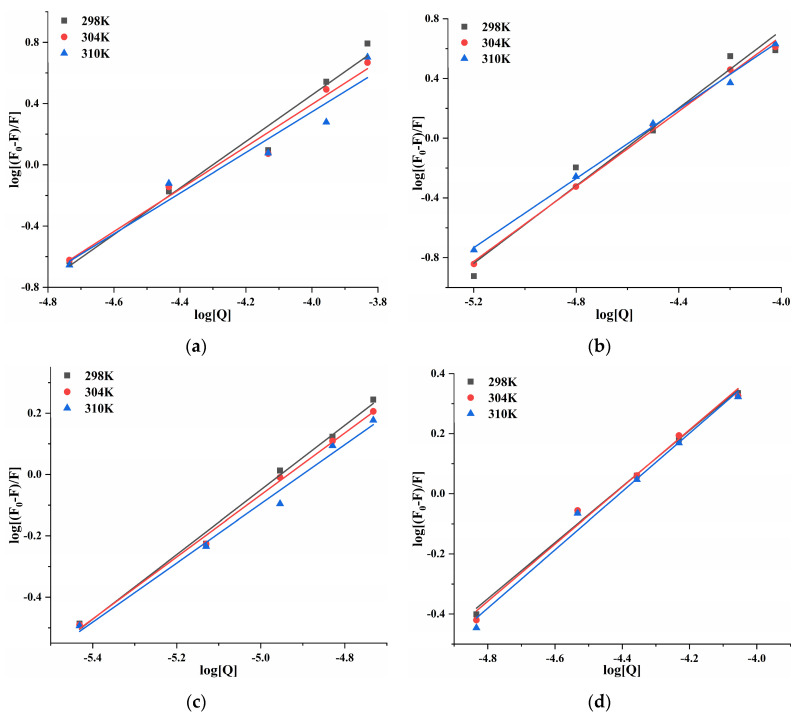
Double logarithmic curve of (**a**) THPP (**b**) TCPP (**c**) TAPP (**d**) Cu-TCPP on PL.

At 298, 304, and 310 K, the log[(*F*_0_ − *F*)/*F*] and log[Q] of four porphyrin compounds with PL showed a good linear relationship (Figure 7). *K_a_* and *n* can be calculated from the intercept and the slope of the curve. The *n* value was close to 1 (Table 2), indicating that THPP, TCPP, TAPP, and Cu-TCPP had only one or one type of binding site with PL, which demonstrated congruence with the results of inhibition kinetics. Four porphyrin compounds had good binding ability to PL. The *K_a_* values of THPP, TCPP, and TAPP decreased significantly with increasing temperature, indicating that the higher the temperature, the worse the stability of the three porphyrin compounds-PL complexes. The *K_a_* value of Cu-TCPP increased gradually with increasing temperature, indicating that the binding ability was enhanced. At 310 K, the binding constants and the binding abilities of four porphyrin compounds were THPP > TCPP > TAPP > Cu-TCPP, which was consistent with the order of inhibitory ability.

The relevant thermodynamic parameters were calculated by Equations (6) and (7), and the Van’t Hoff curves of four porphyrin compounds and enzymes were obtained:

(6) $$\ln K_{a}=-\frac{\Delta H^{0}}{RT}+\frac{\Delta S^{0}}{R},$$

(7) $$\Delta G^{0}=\Delta H^{0}-T\Delta S^{0}=-RT\ln K_{a},$$

**Figure 8:**
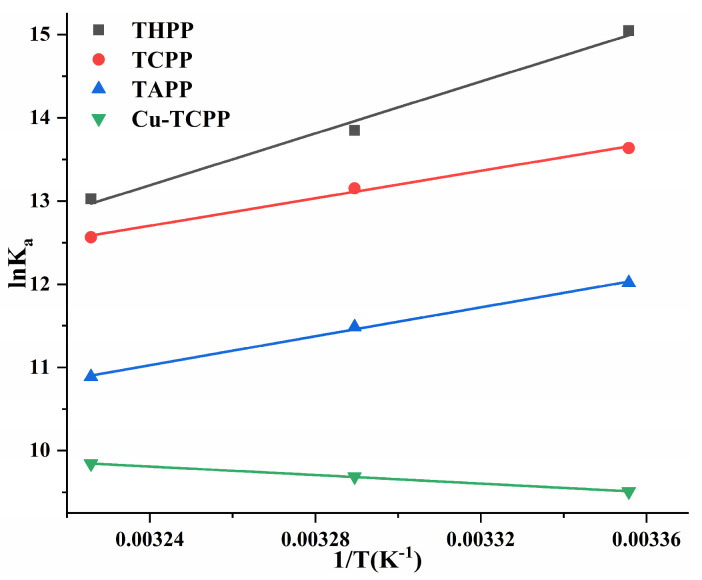
Van’t Hoff curves of four porphyrin compounds on PL.

Using ln*K_a_* ~ 1/*T* as the Van’t Hoff curves of four porphyrin compounds and PL (Figure 8). The ΔG^0^ < 0 of four porphyrin compounds (Table 3) indicated that the binding of PL to THPP, TCPP, TAPP, and Cu-TCPP was spontaneous. In the formation of PL-THPP complex, PL-TCPP complex and PL-TAPP complex, ΔH^0^ < 0 and ΔS^0^ < 0, indicating that THPP, TCPP, and TAPP were mainly dominated by hydrogen bonds and van der Waals forces to bind to PL. In the formation of PL-Cu-TCPP complex, ΔH^0^ > 0 and ΔS^0^ > 0, indicating that Cu-TCPP was mainly bound to PL by a heat-absorbing reaction driven by hydrophobic forces.

### 2.5. Synchronous Fluorescence Analysis of the Pancreatic Lipase-Four Porphyrin Compounds Interaction

**Figure 9:**
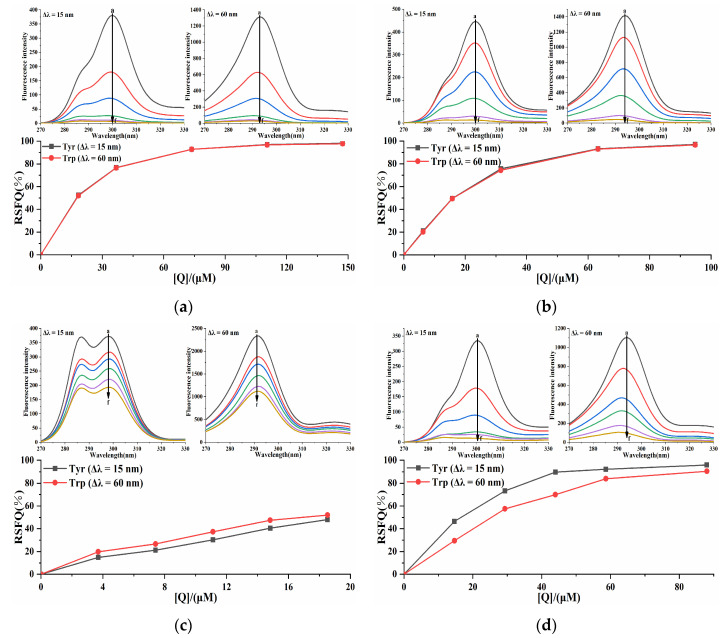
Synchronous fluorescence spectrum of (**a**) THPP (**b**) TCPP (**c**) TAPP (**d**) Cu-TCPP on PL. (**a**) c(THPP) = 0, 18.42, 36.84, 73.67, 110.51, 147.34 μM; (**b**) c(TCPP) = 0, 6.32, 15.81, 31.61, 63.23, 94.84 μM; (**c**) c(TAPP) = 0, 3.70, 7.41, 11.11, 14.82, 18.52 μM; (**d**) c(Cu-TCPP) = 0, 14.67, 29.33, 44.00, 58.66, 88.00 μM; for curves a→f, respectively.

To elucidate the influences of four porphyrin compounds on the microenvironment of amino acid residues in PL, synchronous fluorescence spectroscopy was used to stabilize Δλ at 15 nm and 60 nm to reflect the characteristic fluorescence information of tyrosine (Tyr) and tryptophan (Trp) residues. With the increase in the concentration of four porphyrin compounds, the fluorescence intensity of tyrosine and tryptophan residues of the system gradually decreased (Figure 9), indicating that four porphyrin compounds interacted with PL, which demonstrated congruence with the previous fluorescence quenching results. With the increase in the concentration of THPP, TCPP, and Cu-TCPP, the maximum emission wavelengths of Tyr and Trp residues were blue-shifted, indicating that THPP, TCPP, and Cu-TCPP induced the decrease in polarity around Tyr and Trp residues and the increase in hydrophobicity during the binding process with PL, resulting in less exposure of amino acid residues to solvents. The three porphyrin compounds could change the conformation of PL. When the concentration of TAPP increased, the maximum emission wavelengths of Tyr and Trp were not significantly red-shifted or blue-shifted, indicating that TAPP basically did not change the microenvironment of Tyr and Trp in PL and had little effect on the polarity and hydrophobicity around these two amino acid residues. In order to compare the extent of contribution of Tyr and Trp residues to the interaction between four porphyrin compounds and PL, the RSFQ was calculated by Formula (8). The RSFQ values of THPP and TCPP at Tyr and Trp residues were very close (Figure 9), indicating that THPP and TCPP bound to the vicinity of both Tyr and Trp residues. The RSFQ value of TAPP at Trp was higher than that at Tyr, indicating that the contribution of Trp residue was greater in the endogenous fluorescence quenching effect of TAPP on PL, that is, TAPP was closer to Trp residue [35]. The RSFQ value of Cu-TCPP at Tyr was higher than that at Trp, indicating that the contribution of Tyr residue was greater in the endogenous fluorescence quenching effect of Cu-TCPP on PL, that is, Cu-TCPP was closer to Tyr residue.

(8) $$RSFQ=1-\frac{F}{F_{0}},$$

In the formula, *F*_0_ and *F* have the same meaning as in Formula (3).

### 2.6. Three-Dimensional Fluorescence Analysis of the Pancreatic Lipase-Four Porphyrin Compounds Interaction

**Figure 10:**
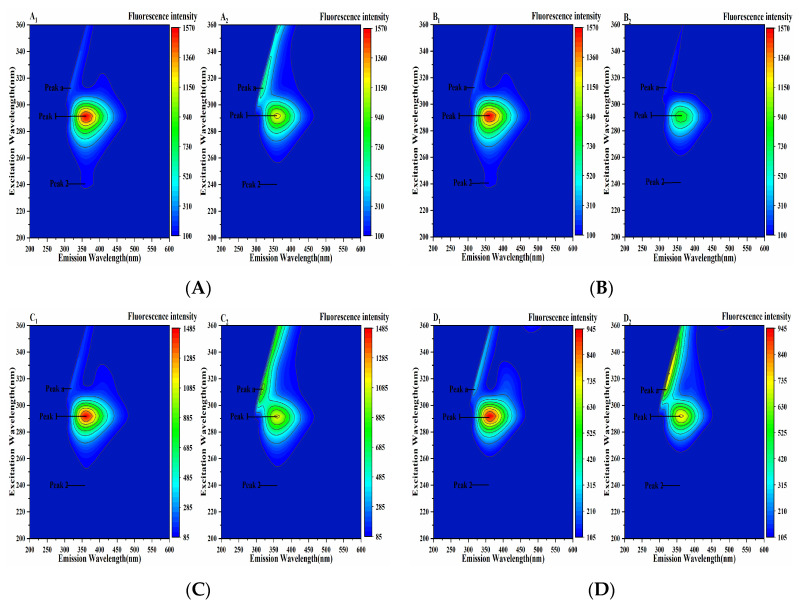
Three-dimensional fluorescence spectrum of the interaction between (**A**) THPP (**B**) TCPP (**C**) TAPP (**D**) Cu-TCPP and PL c(THPP) = 3.68 μM; c(TCPP) = 6.32 μM; c(TAPP) = 7.41 μM; c(Cu-TCPP) = 11.73 μM. (**A_1_**–**D_1_**) respective PL. (**A_2_**–**D_2_**) respective PL-porphyrin compound complex.

Three-dimensional fluorescence spectroscopy can intuitively provide information on protein structure changes. Peak a is the Rayleigh scattering peak, Peak 1 represents the fluorescence information of Tyr and Trp residues, and Peak 2 shows the characteristic peak of the polypeptide chain skeleton conformation (Figure 10). It could be seen from Figure 10 that the fluorescence intensity of Peak 1 and Peak 2 decreased after the addition of four porphyrin compounds, indicating that the addition of THPP, TCPP, TAPP and Cu-TCPP quenched the fluorescence of Tyr and Trp and changed the conformation of PL. It was further speculated that the decrease in fluorescence intensity of Peak 1 could be due to the formation of PL-porphyrin compound complex, which weakened the fluorescence emission of Tyr and Trp residues of PL. The decrease in fluorescence intensity of Peak 2 might be due to the instability of PL polypeptide chain caused by the binding of four porphyrin compounds to PL [36], which changed its hydrophobic microenvironment and led to the change in PL conformation.

### 2.7. FT-IR Spectroscopy Analysis of the Pancreatic Lipase-Four Porphyrin Compounds Interaction

The effects of four porphyrin compounds on the secondary structure of PL were further studied by FT-IR spectroscopy. The amide I band (1700–1600 cm^−1^, mainly C=O stretching) and the amide II band (1600–1500 cm^−1^, mainly C-N stretching) are important spectral bands for studying the secondary structure of proteins. Because the regions of amide I band located at 1649–1660, 1615–1637, 1661–1680, 1638–1648, and 1681–1692 cm^−1^ after curve fitting represent α-helix, β-sheet, β-turn, random coil and β-antiparallel, respectively [37], and the signal intensity is high, it is more widely evaluated than the amide II band.

The amide I bands in the FT-IR spectra of PL and PL-porphyrin compound complexes were deconvolution and peak fitting to obtain the fitting Figure S1. The percentage of each secondary structure calculated after fitting is listed in Table 4. After the addition of four porphyrin compounds, the proportion of α-helix and β-turn decreased, and the proportion of β-sheet and random coil increased. The decrease in α-helix ratio might be due to the combination of four porphyrin compounds and amino acid residues of PL polypeptide chain, which destroyed the hydrogen bond network between protein helix structures, led to the unfolding and instability of PL structure, and changed the surface hydrophobicity. At the same time, it might affect the formation of active sites or hinder the binding of substrates to PL, thus affecting the stability and activity of PL. The increase in β-sheet ratio might be due to the rearrangement of PL secondary structure induced by four porphyrin compounds and the influence of protein folding. The changes in the proportion of the above secondary structures indicated that the interaction between THPP, TCPP, TAPP, Cu-TCPP, and PL might destroy the hydrogen bond network of the enzyme, change the conformation of the enzyme and the structure of the active center and cause the rearrangement of the secondary structure, thereby affecting the formation of active sites or hindering substrate binding, reducing the stability and catalytic activity of PL.

### 2.8. Molecular Docking Analysis of Three Porphyrin Compounds with Pancreatic Lipase

**Figure 11:**
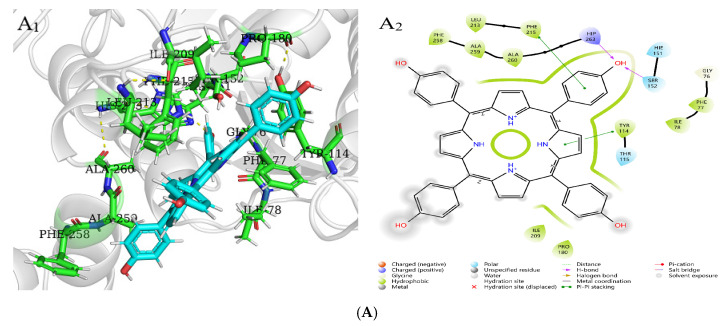

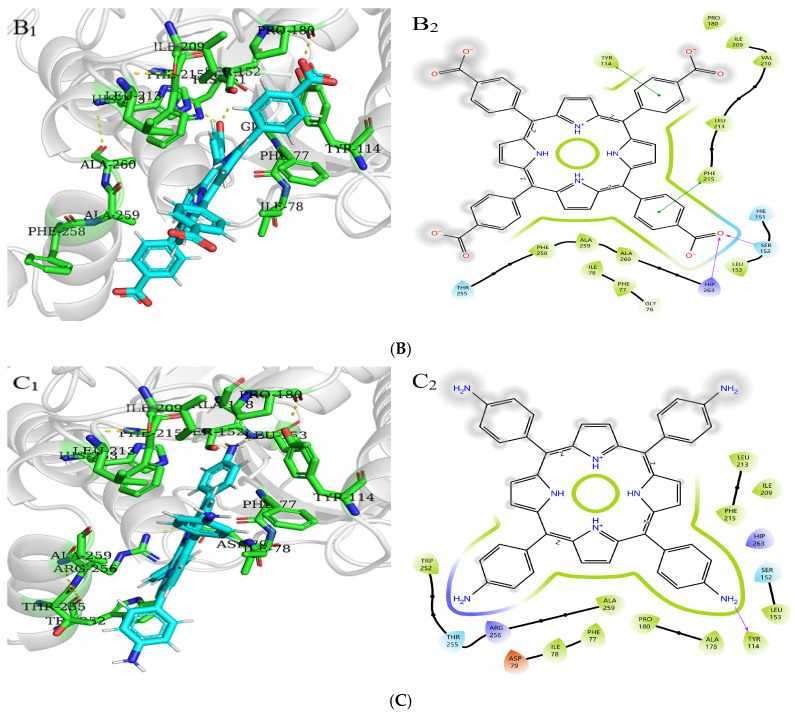
Molecular docking of (**A**) THPP (**B**) TCPP (**C**) TAPP to PL. (**A_1_**–**C_1_**) respective predicted best binding mode. (**A_2_**–**C_2_**) respective 2D diagram.

Molecular docking is used to predict the interaction modes and binding forces between macromolecules and small molecules. It could be seen from Figure 11 that the hydrophobic residues LEU213, PHE215, ALA259, PHE77, ILE78, PRO180, and ILE209 of PL surrounded THPP, TCPP, and TAPP to form hydrophobic interactions. The phenolic hydroxyl group of THPP formed a hydrogen bond with the PL residues HIP263 and SER152, respectively. THPP produced π–π stacking and hydrophobic interactions with the residues PHE215 and TYR114 and was also adjacent to the hydrophobic residues PHE258 and ALA260 to form a hydrophobic force. The carbonyl oxygen atom of the carboxyl group of TCPP formed a hydrogen bond with the residues HIP263 and SER152, respectively. TCPP had π–π stacking and hydrophobic interactions with the residues PHE215 and TYR114 and was also adjacent to the hydrophobic residues VAL210, PHE258, ALA260, and LEU153 to form a hydrophobic force. The amino group of TAPP formed a hydrogen bond with the residue TYR114, which was adjacent to the hydrophobic residues TRP252, ALA178, and LEU153 to form a hydrophobic force. The N-terminal domain of PL has an encapsulated catalytic triad, consisting of SER152, ASP176, and HIS263 [38], which is the active site for substrate hydrolysis. There are three main amino acid residues involved in the catalytic process, and PHE77, HIS151, and PHE215 are potential substrate binding or inhibition sites. SER152 is usually hidden and protected. When the conformation of PL changes, the active catalytic site is exposed, resulting in the loss of PL activity. Orlistat is inhibited by binding to SER152 of PL at the active site [39]. THPP and TCPP interacted with SER152 and HIP263 through hydrogen bonds and interacted with TYR114 and PHE215 through electrostatic forces. TAPP interacted with TYR114 and TRP252 through hydrogen bonding and a hydrophobic interaction, respectively. THPP, TCPP, and TAPP interacted with PHE77 through hydrophobic interactions. This was also consistent with the results of multi-spectral analysis, which confirmed that the interaction between THPP, TCPP, and TAPP, and PL amino acid residues led to conformational transition. In addition, these results indicate that hydrogen bonds played an important role in the interaction of three porphyrin compounds with PL amino acid residues.

XP GScore can be used to evaluate the binding affinity. The smaller the value, the more stable the binding performance of macromolecules and small molecules. As shown in Table 5, the docking scores of PL with THPP, TCPP, TAPP were −3.49, −2.88, −2.62 kcal/mol, respectively. The binding stability was as follows: THPP > TCPP > TAPP, which had the same order with the inhibitory ability.

Hydrogen bonds played a crucial role in regulating the binding stability of THPP, TCPP, TAPP, and PL. The order of inhibitory activity and binding stability was THPP > TCPP > TAPP. Hydroxyl (-OH) had a significant effect on enhancing PL inhibitory activity. The hydroxyl group (-OH) of THPP could form hydrogen bonds with PL amino acid residues, which directly contributed to the binding affinity. The electron-donating effect of hydroxyl groups helped to stabilize the electron cloud balance of porphyrin macrocycles and increased their electron density, resulting in enhanced stability of π–π stacking. The synergistic effect of the two significantly improved the stability of the PL-THPP complex, thereby giving THPP the strongest inhibitory activity. The carboxyl group (-COOH) of TCPP exhibited different electronic effects. The electron-withdrawing property of the carboxyl group destroyed the electron cloud balance of the porphyrin macrocycle, resulting in a relative decrease in its electron density. This weakened the stability of the π–π stacking, thereby reducing the stability of the PL-TCPP complex, showing that its binding stability and inhibitory activity were lower than that of THPP. Due to the small number of hydrogen bonds formed with PL, TAPP had the lowest absolute value of binding energy and the weakest PL inhibitory activity. In summary, the electronic properties (electron-donating or electron-withdrawing) of the peripheral substituents of porphyrin compounds will also affect their binding stability with PL. The hydroxyl group (-OH) is the dominant group to enhance the PL inhibitory activity due to its ability to form hydrogen bonds and electron donor, while the electron-withdrawing of carboxyl group (-COOH) may have an adverse effect.

## 3. Materials and Methods

### 3.1. Materials

TCPP, lipase (porcine pancreas), orlistat, triton x-100, and TRIS were purchased from Shanghai Yuanye Co., Ltd., Shanghai, China. THPP, TAPP, and Cu-TCPP were purchased from Shanghai Macklin Co., Ltd., Shanghai, China. 4-Nitrophenyl laurate and chromatographic methanol were purchased from Shanghai Aladdin Reagent Co., Ltd., Shanghai, China.

### 3.2. Enzymatic Inhibition Studies

#### 3.2.1. Pancreatic Lipase Inhibition Assays

A total of 250 mg pancreatic lipase was dissolved in 50 mL ultrapure water. After centrifugation at 5000 r/min for 10 min, the supernatant was taken. Then, 80 mg of 4-nitrophenyl laurate was dissolved in 100 mL of 5 mmol/L sodium acetate solution (pH 5.0, containing 1% triton x-100), and dissolved in a boiling water bath. After cooling to room temperature, it was set aside. Appropriate amounts of TCPP, THPP, Cu-TCPP, and TAPP powders were dissolved in DMSO (final concentrations of 2%, 2%, 5%, and 10%, respectively), and then diluted to different concentrations by adding chromatographic methanol and stored in brown reagent bottles in the dark. The blank sample solutions were chromatographic methanol solutions containing 2%, 5%, and 10% DMSO. Orlistat was prepared from the blank sample solution.

The activity of PL was determined by referring to the method and making appropriate modifications [40]. A total of 200 μL of different concentrations of four porphyrin compounds, 500 μL of 4-nitrophenyl laurate (0.80 mg/mL), 400 μL of Tris-HCl buffer (pH 8.2, 0.1 mol/L), and 300 μL of pancreatic lipase (5 mg/mL) were reacted in an incubator at 37 °C for 2 h. The reaction solution was centrifuged at 12,000 rpm for 5 min, and 200 μL of the supernatant was added to a 96-well plate. The absorbance at 420 nm was measured, and orlistat was used as a positive control. $\mathrm{inhibition}\;\mathrm{rate}\%=\left(1-\left(A_{1}-A_{2}\right)/\left(A_{3}-A_{4}\right)\right)\times 100$, where *A*_1_ is the sample group, and *A*_2_ is the sample control group using Tris-HCl buffer instead of enzyme solution, and *A*_3_ is the blank group using blank sample solution instead of sample solution, and *A*_4_ is the blank control group using blank sample solution and Tris-HCl buffer instead of sample solution and enzyme solution, respectively.

#### 3.2.2. Mode of Pancreatic Lipase Inhibition

The concentration of substrate 4-nitrophenyl laurate was fixed at 0.80 mg/mL, and the concentration of PL was changed (5, 7.5, 10, 12.5 mg/mL). By analyzing the relationship between enzyme concentration and reaction rate, a fitting straight line was drawn to determine whether it was reversible.

The concentration of PL was fixed at 5 mg/mL, and the concentration of the substrate 4-nitrophenyl laurate was changed (0.40, 0.80, 1.20, 1.60 mg/mL). By analyzing the relationship between the concentration of different substrates and the reaction rate of the enzyme, the Lineweaver–Burk double reciprocal curve was plotted to determine the inhibition type and calculate the inhibition constant.

#### 3.2.3. Study on Stability In Vitro

Temperature stability study. Sample pre-treatment: 1000 μL samples were bathed in water at different temperatures (30, 40, 50, 60, 70, 80, 90, 100 °C) for 20 min, and cooled rapidly.

Acid-base stability study. Sample pre-treatment: 1000 μL samples were treated with buffers of different pH (2.0, 3.0, 4.0, 5.0, 6.0, 7.0, 8.0, 9.0, and 10.0) for 20 min, and then the pH was adjusted back to 6.9–7.3.

PL inhibition experiments were the same as Section 3.2.1. A total of 200 μL pre-treated four porphyrin compounds, 500 μL of 4-nitrophenyl laurate (0.80 mg/mL), 400 μL of Tris-HCl buffer (pH 8.2, 0.1 mol/L), and 300 μL of pancreatic lipase (5 mg/mL) were reacted in an incubator at 37 °C for 2 h. The reaction solution was centrifuged at 12,000 rpm for 5 min, and 200 μL of the supernatant was added to a 96-well plate. The absorbance at 420 nm was measured. The temperature stability range was determined by comparing the inhibition rate of the samples treated at different temperatures. The range of acid-base stability was determined by comparing the changes in inhibition rate.

### 3.3. Fluorescence Analysis of the Pancreatic Lipase-Inhibitor Interaction

The method [41] of Li et al. was referred to and appropriate changes were made. A series of concentrations of porphyrin compounds were mixed with pancreatic lipase (5 mg/mL) at a volume ratio of 1:1 and incubated at 298, 304, and 310 K for 5 min. The excitation wavelength of the Hitachi fluorescence spectrometer F-7000 (Shimadzu, Japan) was set to 280 nm, the slit width was set to 5 nm, and the scanning range was set to 300–500 nm. In order to eliminate the influence of ‘internal filtering effect’ [42], the following formula is used for correction. $F_{cor}=F_{obs}e^{\left(A_{ex}+A_{em}\right)/2}$, where *F_cor_* and *F_obs_* are the corrected fluorescence intensity and the experimentally measured fluorescence intensity, and *A_ex_* and *A_em_* are the UV absorption values of the system at the excitation and emission wavelengths, respectively.

### 3.4. Synchronous Fluorescence Analysis of the Pancreatic Lipase–Inhibitor Interaction

At 298 K, in the synchronous scanning mode, the excitation and emission wavelength intervals (Δλ) were set to 15 nm and 60 nm, and the fluorescence titration conditions were the same as above. The synchronous fluorescence spectra of the reaction system at 200–400 nm were recorded.

### 3.5. Three-Dimensional Fluorescence Analysis of the Pancreatic Lipase-Inhibitor Interaction

At 298 K, in the three-dimensional scanning mode, the excitation and emission wavelength range were set to 200–600 nm. The fluorescence titration conditions were the same as above. The three-dimensional fluorescence spectrum of the reaction system was detected.

### 3.6. FT-IR Spectroscopy Analysis of the Pancreatic Lipase-Inhibitor Interaction

The pancreatic lipase-porphyrin compound solution with a molar ratio of 1:2 was placed in a freeze-centrifugal concentrator Auto R1-plus (Beijing Jiaimu, Beijing, China). The dried solid samples were obtained and mixed with dried KBr, ground, and pressed into tablets, and then detected by Nicolet IS-50 (ThermoFisher, Waltham, MA, USA) instrument. The scanning range was 400–4000 cm^−1^ and the resolution was 4 cm^−1^. Peakfit V4.12 software was used to fit the amide I region (1600–1700 cm^−1^) of pancreatic lipase.

### 3.7. Molecular Docking

The possible binding modes of three representative porphyrin compounds (THPP, TCPP, and TAPP) to pancreatic lipase were studied by Schrödinger software Maestro. The crystal structure of pancreatic lipase (PDB ID: 1LPB [43]) was obtained from the RCSB PDB protein database, and the protein crystals were processed using the Protein Preparation Workflow module of Schrödinger software. The 2D sdf structure files of the three porphyrin compounds were downloaded from PubChem, and the ligands were prepared using the LigPrep module of Schrödinger software. XP Precision Docking was used to dock the processed ligands with the active site of the obtained pancreatic lipase. PyMOL software was used to further visualize the possible binding mode of pancreatic lipase with porphyrin compounds.

### 3.8. Statistical Analysis

Parallel experiments were conducted three times. The data were plotted and statistically analyzed by Origin 2018 and Excel 2019 software. The chart data were expressed as mean ± standard deviation.

## 4. Conclusions

In this paper, the interaction mechanism between four porphyrin compounds (THPP, TCPP, TAPP, Cu-TCPP) and PL was studied by enzyme inhibition kinetics, multi-spectral technology, and molecular simulation technology. The results showed that the four porphyrin compounds exhibited better inhibition ability of enzyme activity. The IC_50_ values were 97.49, 100.10, 236.40, and 248.70 μM, respectively. The inhibitory ability of PL was as follows: THPP > TCPP > TAPP > Cu-TCPP. Four porphyrin compounds reversibly inhibited the activity of PL by UC. The order of *K_i_* values was consistent with the order of IC_50_ values. Four porphyrin compounds showed good thermal stability in a short time and had a wide range of pH tolerance. THPP quenched the endogenous fluorescence of PL in the form of static quenching and dynamic quenching. TCPP, TAPP, and Cu-TCPP reduced the fluorescence intensity of PL in the form of static quenching. Four porphyrin compounds spontaneously bound to PL, and there was only one binding site. Hydrogen bonds and van der Waals forces were the main driving forces for the binding of THPP, TCPP, and TAPP to PL, while Cu-TCPP was mainly driven by a hydrophobic interaction. THPP, TCPP, and Cu-TCPP could induce the decrease in polarity and increase in hydrophobicity around Tyr and Trp residues, thus altering the conformation of PL, while TAPP had little effect on the polarity and hydrophobicity around Tyr and Trp residues. THPP and TCPP bound to the vicinity of both Tyr and Trp residues. TAPP was closer to Trp residue, and Cu-TCPP was closer to Tyr residue. Four porphyrin compounds reduced the proportion of α-helix and β-turn in the secondary structure of PL, and increased the proportion of β-sheet and random coil, thereby reducing stability and catalytic activity of PL. In the interaction of THPP, TCPP, and TAPP with amino acid residues of PL, the formation of hydrogen bonds played an important role in the binding stability. This study provides a theoretical reference and experimental evidence for porphyrin compounds as potential enzyme inhibitors for the treatment of obesity. Porphyrins and their metal porphyrin compounds are ideal photosensitizers for photodynamic therapy, which are usually non-toxic or have low dark cytotoxicity. We now only analyze the interaction from the mechanism level, which is relatively simple. If the follow-up drug development is carried out, it is necessary to carry out relevant toxicity experiments, and to further study the inhibitory effect and mechanism of porphyrin compounds on PL activity by establishing cell models, animal experiments, and drug clinical trials. The purpose is to obtain the data of biological toxicity, biological activity, and bioavailability in vivo, so as to more truly reflect the lipid-lowering effect of porphyrin compounds in vivo.

## Figures and Tables

**Table 1:** Inhibition kinetic constants of four porphyrin compounds on PL.

System	C (μM)	*K_m_* (μM)	*V_max_* (ΔOD/min)	*K_i_* (μM)
PL-THPP	0	0.6122 ± 0.0093	0.0054 ± 0.0008	37.0317 ± 0.1864
	36.84	0.4652 ± 0.0107	0.0041 ± 0.0009	
	73.67	0.3925 ± 0.0151	0.0035 ± 0.0013	
	147.34	0.2050 ± 0.0075	0.0018 ± 0.0007	
	221.01	0.1332 ± 0.0045	0.0012 ± 0.0004	
	294.68	0.1086 ± 0.0026	0.0009 ± 0.0002	
PL-TCPP	0	0.6122 ± 0.0093	0.0054 ± 0.0008	53.4882 ± 0.1409
	12.65	0.4794 ± 0.0128	0.0042 ± 0.0011	
	31.61	0.4239 ± 0.0092	0.0037 ± 0.0008	
	63.23	0.3108 ± 0.0142	0.0027 ± 0.0012	
	126.45	0.1738 ± 0.0041	0.0015 ± 0.0004	
	189.68	0.1338 ± 0.0029	0.0012 ± 0.0003	
PL-TAPP	0	0.4584 ± 0.0145	0.0040 ± 0.0013	149.8681 ± 0.1952
	118.55	0.2551 ± 0.0037	0.0023 ± 0.0003	
	177.83	0.2096 ± 0.0036	0.0018 ± 0.0003	
	237.11	0.1786 ± 0.0068	0.0016 ± 0.0006	
	266.75	0.1651 ± 0.0029	0.0015 ± 0.0003	
	296.38	0.1545 ± 0.0057	0.0014 ± 0.0005	
PL-Cu-TCPP	0	0.2054 ± 0.0054	0.0055 ± 0.0014	196.1540 ± 0.1310
	29.33	0.1920 ± 0.0049	0.0052 ± 0.0013	
	58.66	0.1643 ± 0.0037	0.0044 ± 0.0009	
	117.33	0.1333 ± 0.0022	0.0036 ± 0.0006	
	175.99	0.1115 ± 0.0046	0.0030 ± 0.0012	
	234.66	0.0988 ± 0.0017	0.0026 ± 0.0005	

**Table 2:** The fluorescence related information of the interaction between four porphyrin compounds and PL were calculated.

System	T	*K_sv_* (×10^4^ L/mol)	*K_q_* (×10^12^ L/mol/s)	n	*K_a_* (×10^5^ L/mol)
PL-THPP	298	1.36 ± 0.02	1.36 ± 0.02	1.52	34.27 ± 0.12
	304	1.17 ± 0.03	1.17 ± 0.03	1.38	10.33 ± 0.08
	310	1.11 ± 0.04	1.11 ± 0.04	1.33	4.54 ± 0.05
PL-TCPP	298	4.60 ± 0.07	4.60 ± 0.07	1.30	8.37 ± 0.07
	304	4.59 ± 0.06	4.59 ± 0.06	1.26	5.15 ± 0.04
	310	4.50 ± 0.06	4.50 ± 0.06	1.16	2.86 ± 0.03
PL-TAPP	298	9.70 ± 0.11	9.70 ± 0.11	1.05	1.66 ± 0.01
	304	8.80 ± 0.06	8.80 ± 0.06	1.01	0.97 ± 0.02
	310	8.17 ± 0.08	8.17 ± 0.08	0.96	0.54 ± 0.01
PL-Cu-TCPP	298	2.36 ± 0.01	2.36 ± 0.01	0.93	0.13 ± 0.01
	304	2.33 ± 0.03	2.33 ± 0.03	0.95	0.16 ± 0.01
	310	2.32 ± 0.03	2.32 ± 0.03	0.97	0.19 ± 0.01

**Table 3:** Thermodynamic parameters of the interaction of four porphyrin compounds with PL.

System	T	ΔH^0^ (kJ/mol)	ΔG^0^ (kJ/mol)	ΔS^0^ (J/mol/k)	R^2^
PL-THPP	298	−129.48 ± 0.11	−37.28 ± 0.17	−309.86 ± 0.12	0.98
	304		−34.99 ± 0.11		
	310		−33.57 ± 0.09		
PL-TCPP	298	−68.59 ± 0.07	−33.79 ± 0.04	−116.60 ± 0.11	0.99
	304		−33.24 ± 0.13		
	310		−32.38 ± 0.08		
PL-TAPP	298	−72.32 ± 0.05	−29.78 ± 0.05	−142.66 ± 0.06	0.99
	304		−29.03 ± 0.07		
	310		−28.06 ± 0.06		
PL-Cu-TCPP	298	21.34 ± 0.04	−23.56 ± 0.08	150.70 ± 0.07	0.99
	304		−24.48 ± 0.05		
	310		−25.37 ± 0.05		

**Table 4:** Secondary structure analysis of PL and PL-porphyrin compound complex.

System	α-Helix (%)	β-Sheet (%)	β-Turn (%)	Random Coil (%)	β-Antiparallel (%)
PL	25.48 ± 0.06	27.31 ± 0.06	23.12 ± 0.03	16.30 ± 0.04	7.78 ± 0.02
PL-THPP	13.96 ± 0.04	37.64 ± 0.05	19.70 ± 0.04	20.38 ± 0.02	8.32 ± 0.01
PL-TCPP	22.26 ± 0.08	37.79 ± 0.12	10.43 ± 0.03	24.70 ± 0.06	4.82 ± 0.01
PL-TAPP	12.81 ± 0.03	36.59 ± 0.07	18.09 ± 0.04	24.25 ± 0.07	8.26 ± 0.02
PL-Cu-TCPP	15.78 ± 0.05	46.44 ± 0.13	13.51 ± 0.03	23.40 ± 0.06	0.87 ± 0.01

**Table 5:** Molecular docking of three porphyrin compounds to PL.

System	XP GScore (kcal/mol)	Key Residues	Hydrogen Bonds
PL-THPP	−3.49	LEU213, PHE215, PHE258, ALA259, ALA260, HIP263, SER152, PHE77, ILE78, TYR114, PRO180, ILE209	HIP263, SER152
PL-TCPP	−2.88	PHE215, LEU213, VAL210, ILE209, PHE258, ALA259, ALA260, HIP263, SER152, LEU153, PHE77, ILE78, TYR114, PRO180	HIP263, SER152
PL-TAPP	−2.62	LEU213, PHE215, ALA259, TRP252, PHE77, ILE78, TYR114, PRO180, ALA178, ILE209, LEU153	TYR114

## Data Availability

Data availability All data included in this study are available upon request by contact with the corresponding author.

## Supplementary Materials

The following supporting information can be downloaded at: https://www.mdpi.com/article/10.3390/molecules30132701/s1, Figure S1: FT-IR spectrum amide I band fitting plot (a) PL (b) PL-THPP (c) PL-TCPP (d) PL-TAPP (e) PL-Cu-TCPP.
