# Supplementary figures and images for: Understanding Anti-Obesity Potential of Four Porphyrin Compounds by Investigating Pancreatic Lipase Inhibition
Source: Molecules. 2025 Jun 23;30(13):2701. doi: 10.3390/molecules30132701 (PMC12251151; doi:10.3390/molecules30132701)

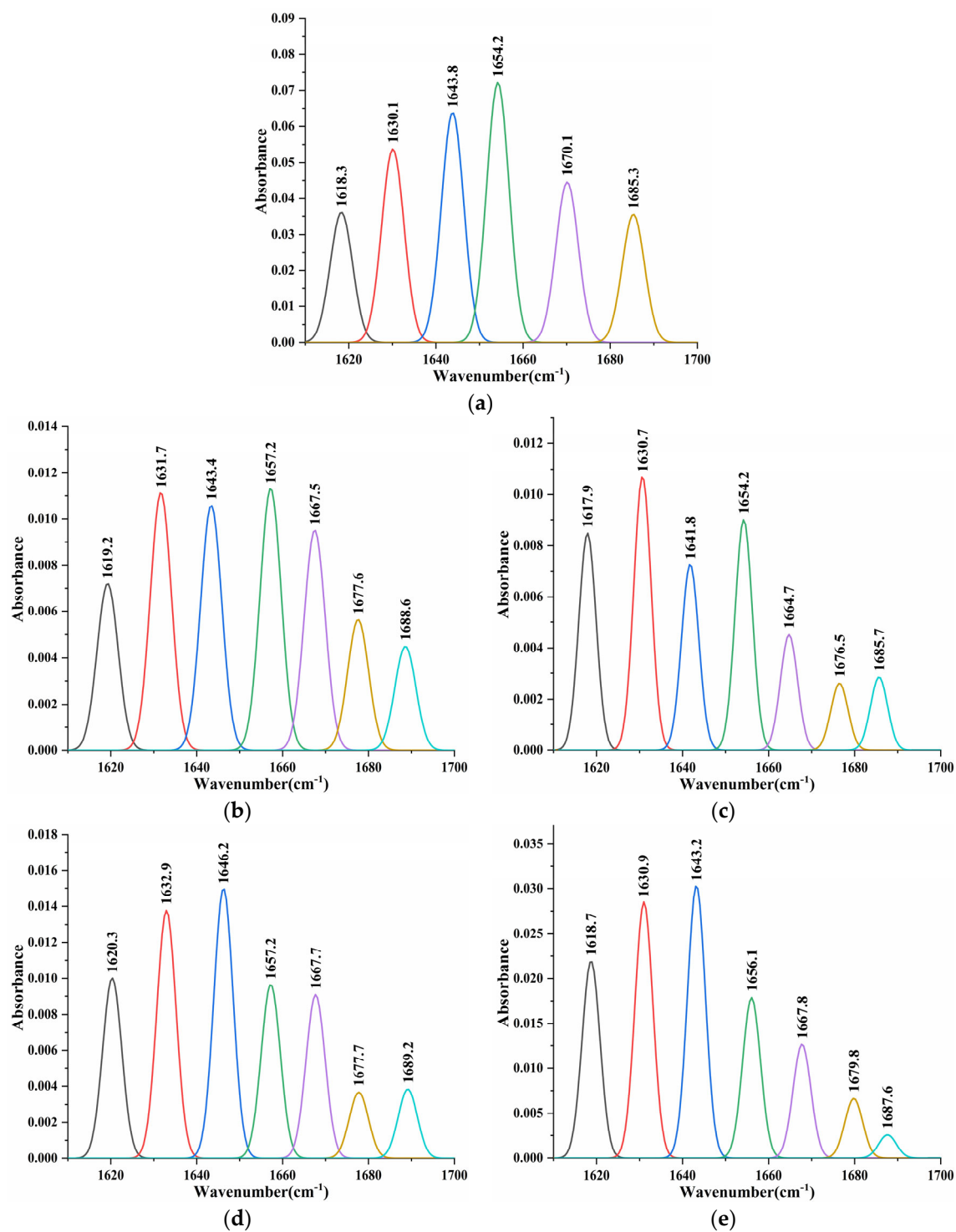

**Figure S1.** FT-IR spectrum amide I band fitting plot (a) PL (b) PL-THPP (c) PL-TCPP (d) PL-TAPP (e) PL-Cu-TCPP.

Supplement: Supplementary file 1 [file molecules-30-02701-s001.zip › molecules-3642698-supplementary.pdf]
